# Supplementary material for: Molecular tuning boosts asymmetric C-C coupling for CO conversion to acetate
Source: Nat Commun. 2024 Apr 29;15:3641. doi: 10.1038/s41467-024-47913-1 (PMC11059391; doi:10.1038/s41467-024-47913-1)
Supplement: Supplementary file 1 — Supplementary Information [file 41467_2024_47913_MOESM1_ESM.pdf]

Molecular Tuning Boosts Asymmetric C-C Coupling for CO Conversion to Acetate

Jie Ding[1,#], Fuhua Li[1,#], Xinyi Ren[2], Yuhang Liu[3], Yifan Li[4], Zheng Shen[2], Tian Wang[5], Weijue Wang[2], Yang-Gang Wang[6], Yi Cui[4], Hongbin Yang[3,*], Tianyu Zhang[7,*] and Bin Liu[1,8,*]

[1]Department of Materials Science and Engineering, City University of Hong Kong, Hong Kong SAR 999077, China

[2]CAS Key Laboratory of Science and Technology on Applied Catalysis, Dalian Institute of Chemical Physics, Chinese Academy of Sciences, Dalian 116023, China

[3]School of Materials Science and Engineering, Suzhou University of Science and Technology, Suzhou 215009, China

[4]Vacuum Interconnected Nanotech Workstation, Suzhou Institute of Nano-Tech and Nano-Bionics, Chinese Academy of Sciences, Suzhou 215123, China

[5]Department of Chemical & Biomolecular Engineering, National University of Singapore, 4 Engineering Drive 4, Singapore 117585, Singapore

[6]Department of Chemistry and Guangdong Provincial Key Laboratory of Catalysis, Southern University of Science and Technology, Shenzhen 518055, China

[7]College of Environmental Science and Engineering, Beijing Forestry University, Beijing 100083, China

[8]Department of Chemistry & Center of Super-Diamond and Advanced Films (COSDAF), City University of Hong Kong, Hong Kong SAR 999077, China

[#]These authors contributed equally.

E-mail: hbyang@dicp.ac.cn (H. Yang), tzhang@bjfu.edu.cn (T. Zhang) and bliu48@cityu.edu.hk (B. Liu)

Table of contents

Supplementary Figures 1-36

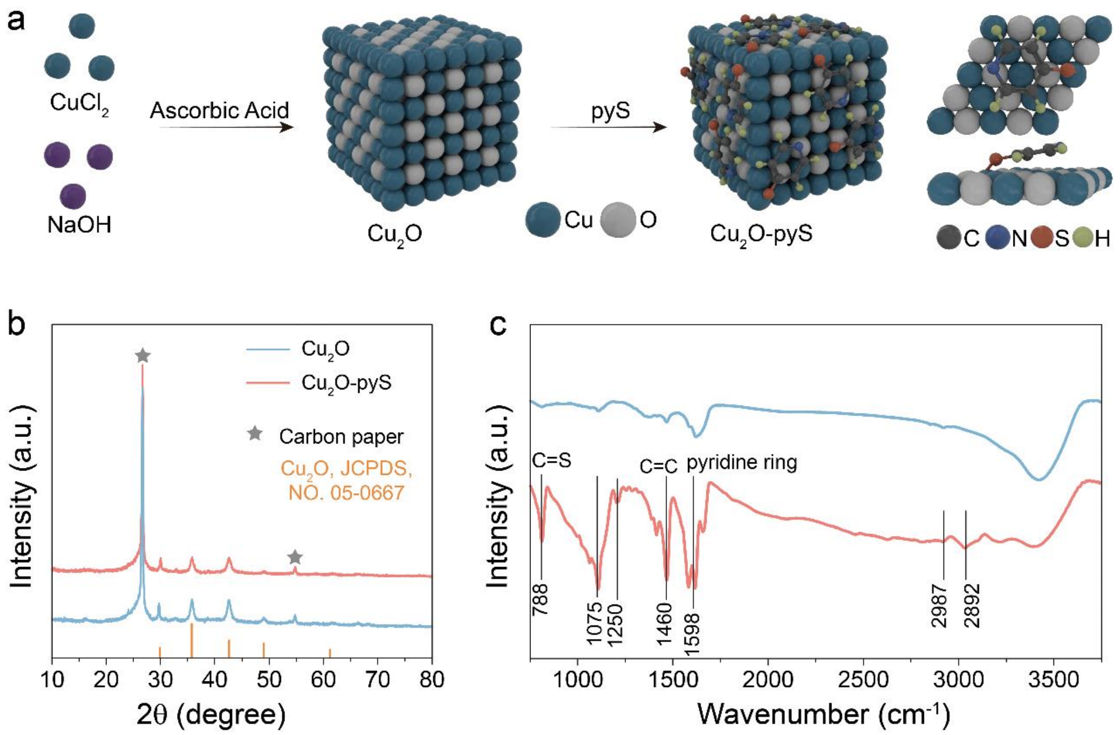

Supplementary Fig. 1 (a) Schematic illustration showing the synthesis step of Cu_2O-pyS. (b) XRD patterns of the as-synthesized Cu_2O nanocubes and Cu_2O-pyS. (c) IR spectra of the as-synthesized Cu_2O nanocubes and Cu_2O-pyS. The peak at 1598 cm^{-1} can be assigned to the stretching vibration of pyridine ring.

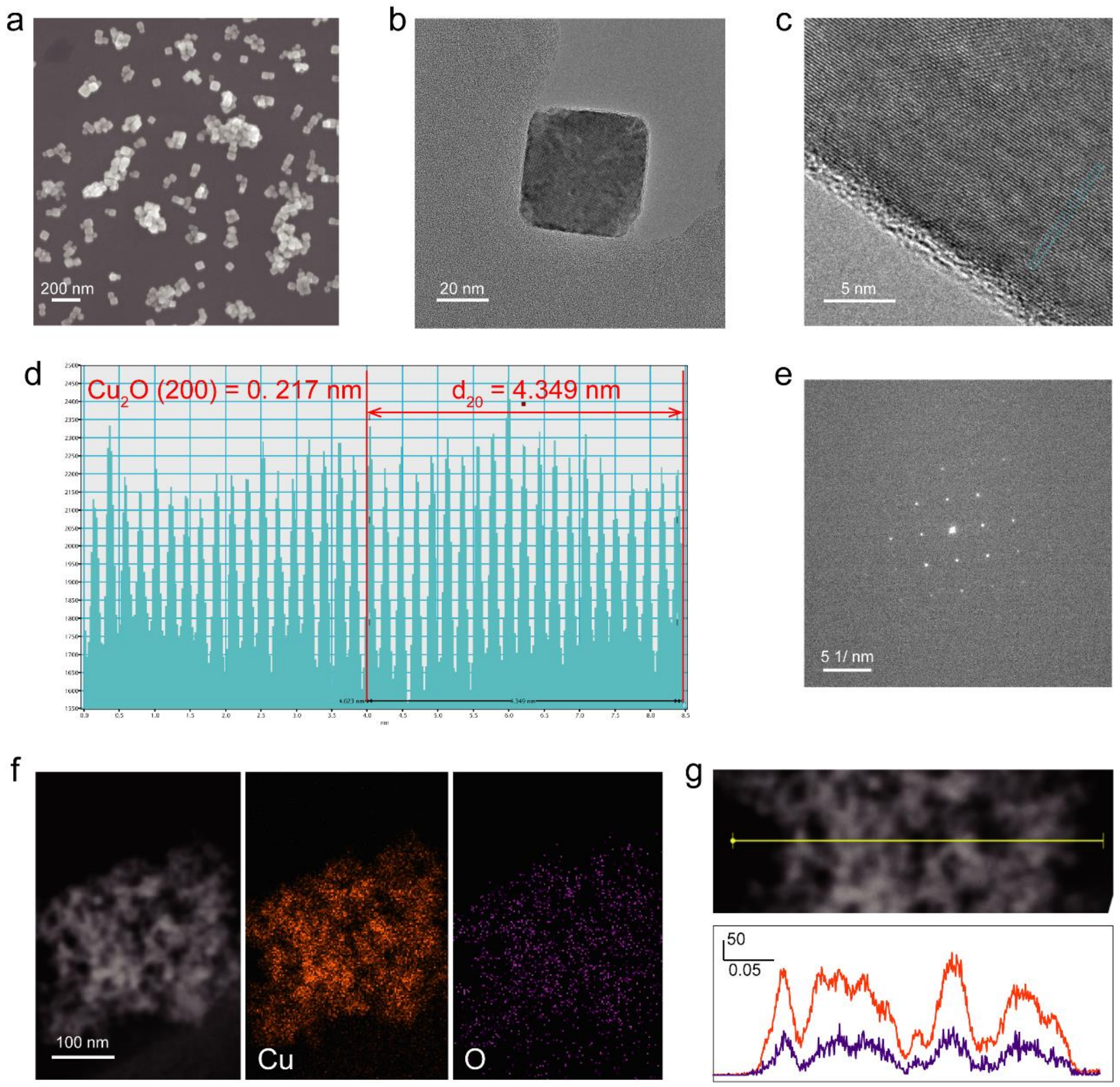

Supplementary Fig. 2 (a) SEM image of Cu_2O nanocubes. (b) TEM image of Cu_2O nanocubes. (c) High-resolution TEM image of Cu_2O nanocubes. (d) Measurement of the lattice spacing of Cu_2O nanocubes. (e) Electron-diffraction pattern of Cu_2O nanocubes. (f) EDS elemental mapping images of Cu_2O nanocubes. (g) EDS line scan of Cu_2O nanocubes. The top image (scale bar: 500 nm) showing the HAADF-STEM image of Cu_2O nanocubes. The bottom image showing the intensity profile of Cu and O along the line as shown in the top.

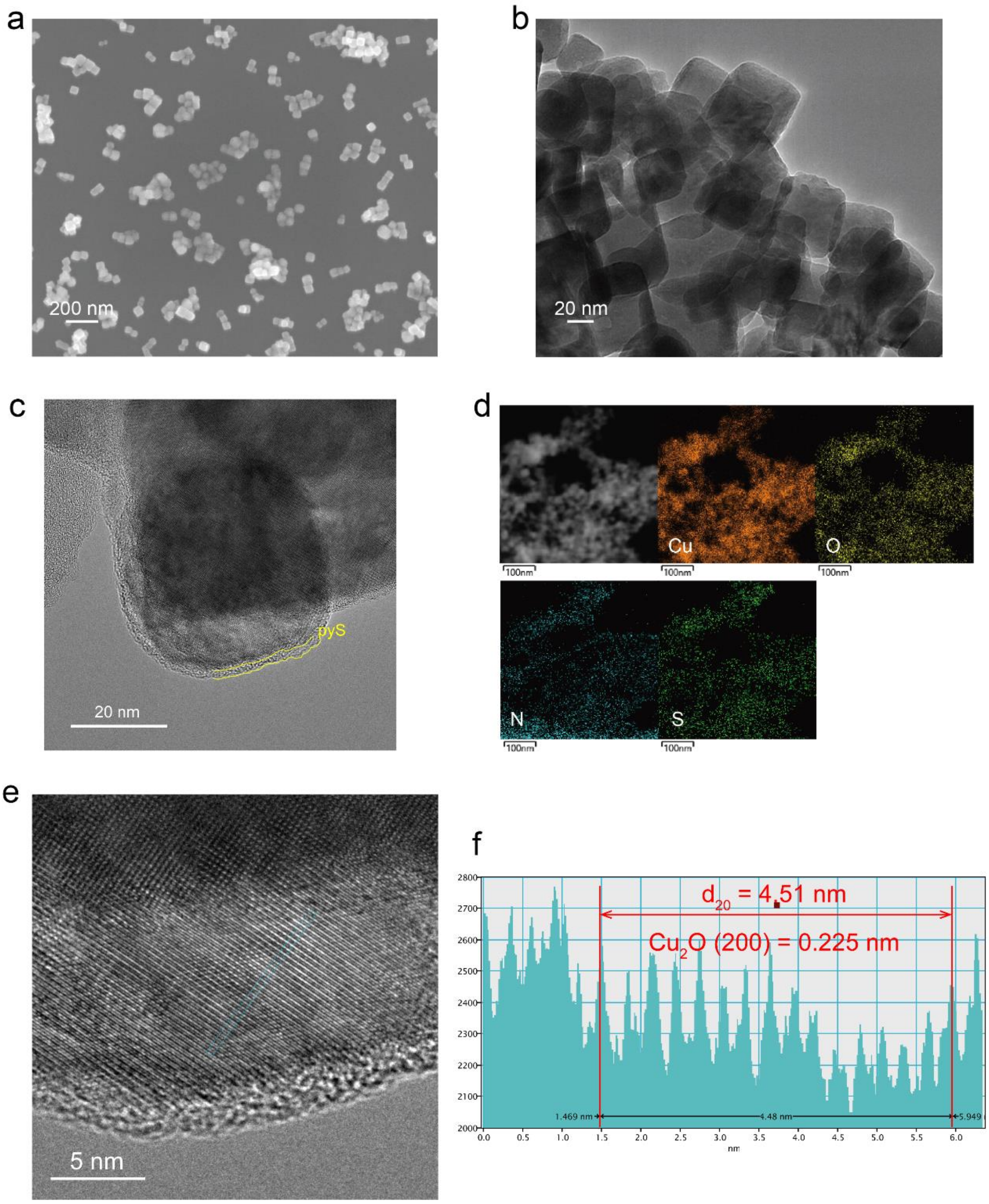

Supplementary Fig. 3 (a) SEM image of Cu_2O-pyS. (b) TEM image of Cu_2O-pyS. (c) High-resolution TEM image of Cu_2O-pyS. (d) EDS elemental mapping images of Cu_2O-pyS. (e) High-resolution TEM image of Cu_2O-pyS showing the lattice spacing. (f) Measurement of the lattice spacing of Cu_2O-pyS.

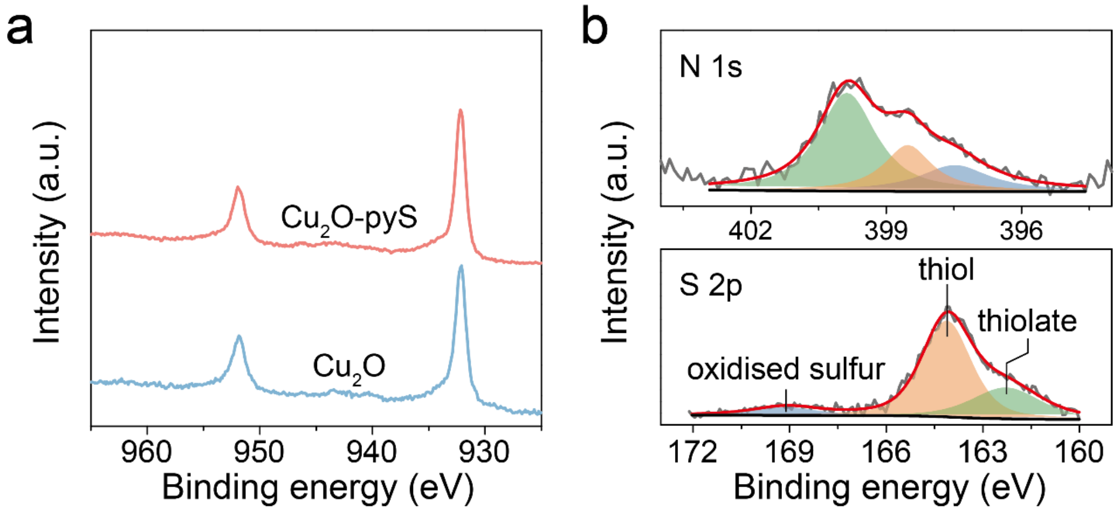

Supplementary Fig. 4 (a) High-resolution Cu 2p XPS spectra of Cu_2O nanocubes and Cu_2O-pyS. (b) High-resolution XPS N 1s XPS spectrum (top) and S 2p XPS spectrum (bottom).

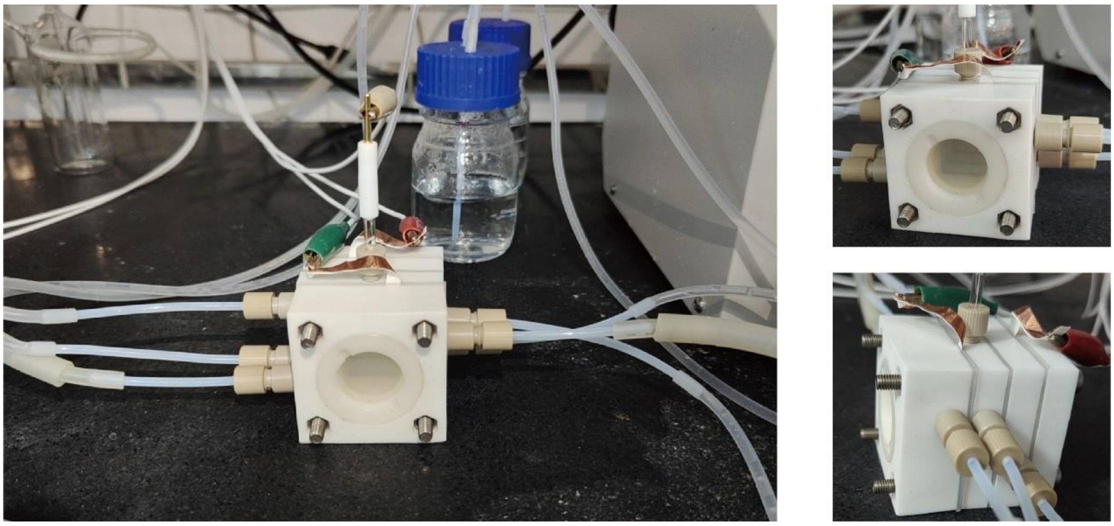

Supplementary Fig. 5 The photograph showing the CO reduction electrolyzer.

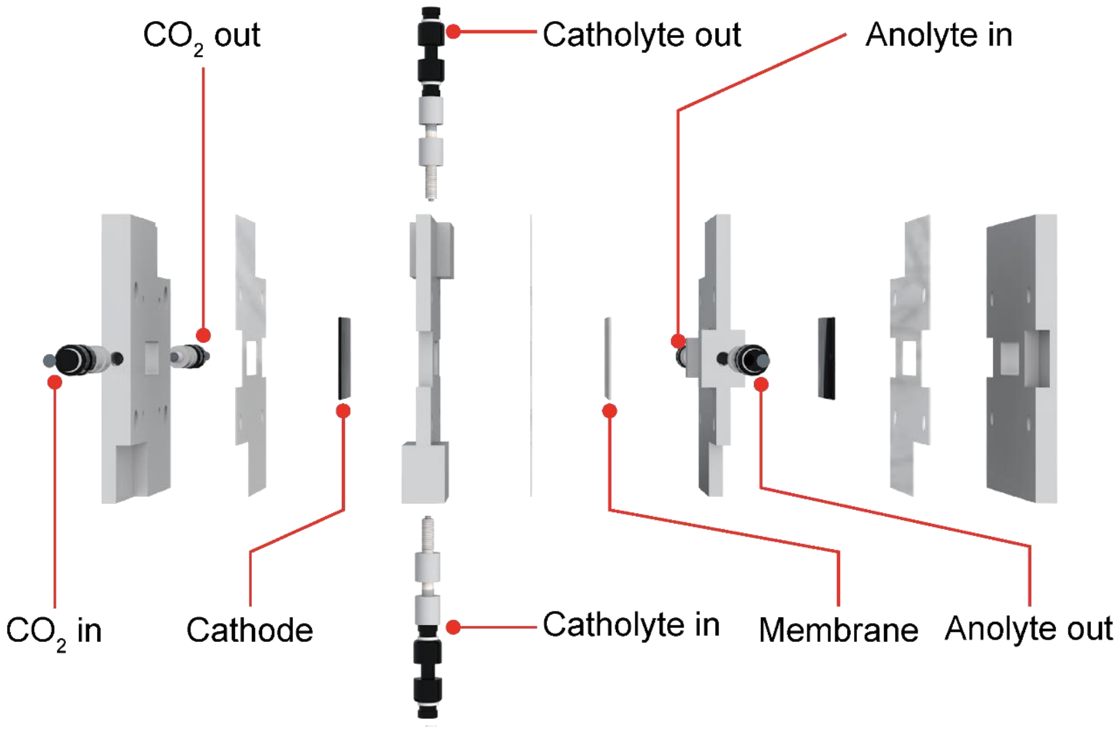

Supplementary Fig. 6 The schematic diagram of the electrochemical flow cell.

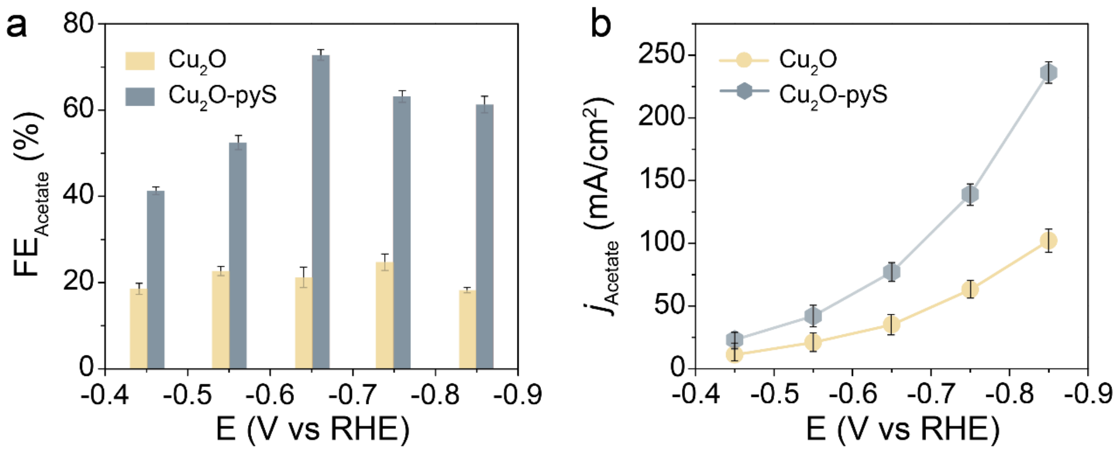

Supplementary Fig. 7 FE (a) and partial current density (b) of acetate over Cu_2O nanocubes and Cu_2O-pyS under various applied potentials. Error bars represent the standard deviation of 3 replicate measurements.

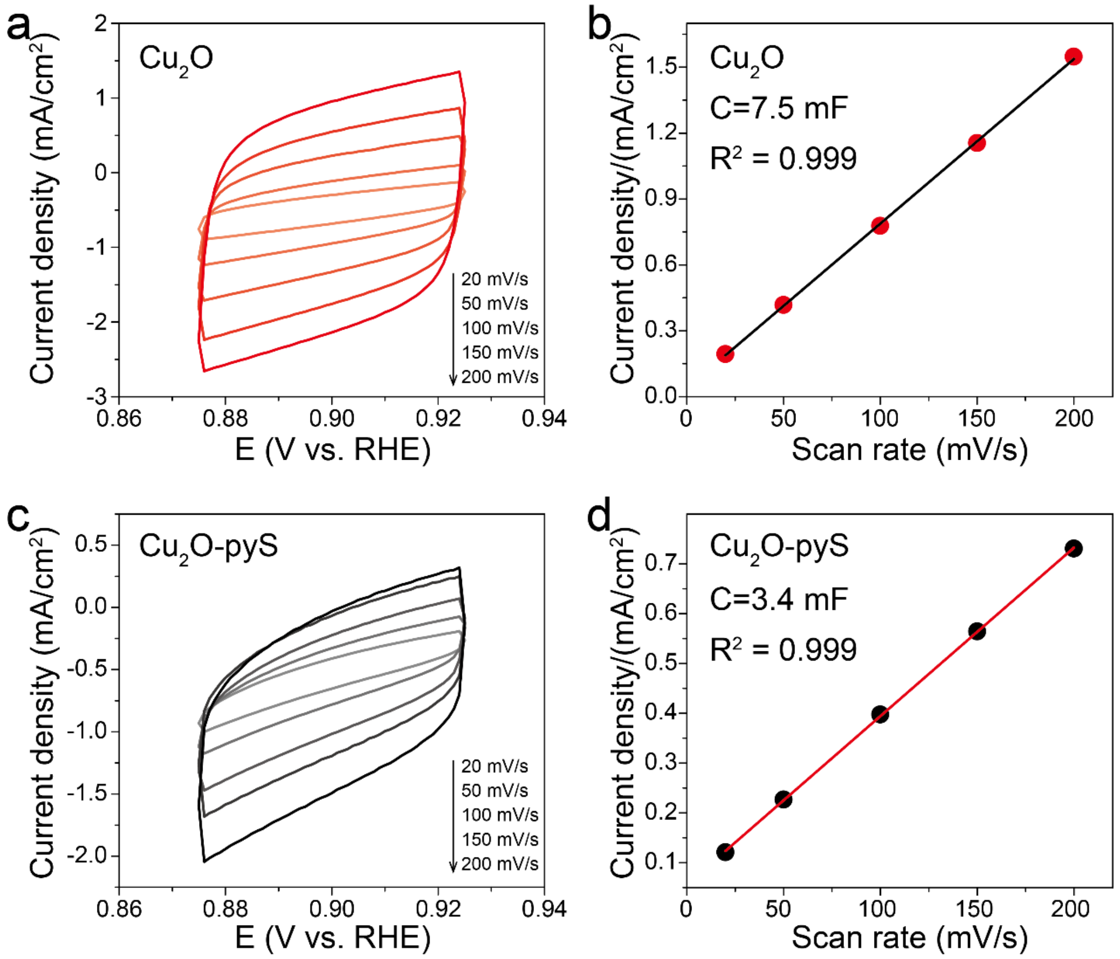

Supplementary Fig. 8 Determination of electrochemically active surface area (ECSA). CV curves of Cu_2O nanocubes (a) and Cu_2O-pyS (c). The corresponding double layer capacitance of Cu_2O nanocubes (b) and Cu_2O-pyS (d).

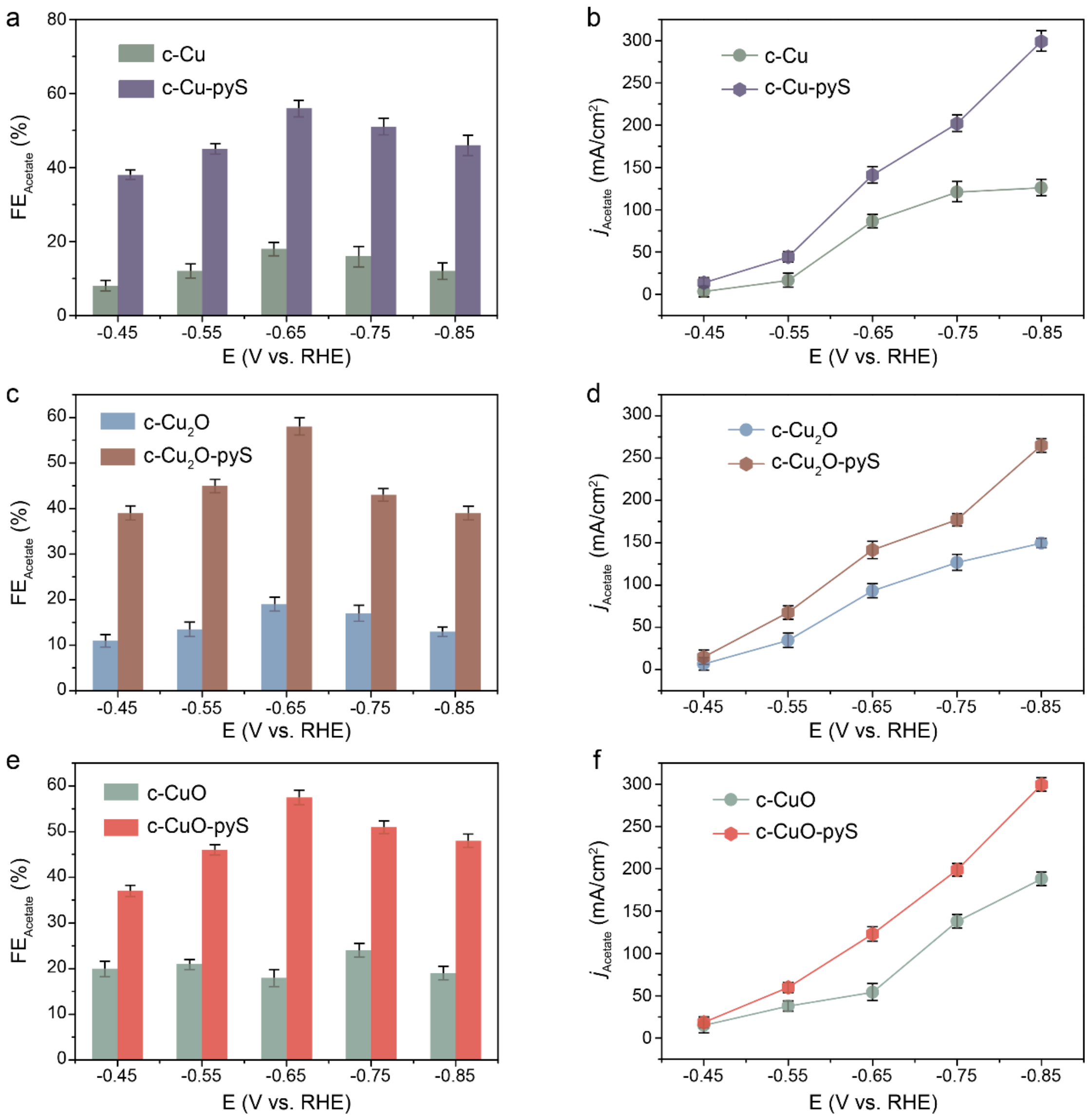

Supplementary Fig. 9 (a) FE of acetate over commercial Cu and c-Cu-pyS. (b) Partial current density of acetate over commercial Cu and c-Cu-pyS. (c) FE of acetate over commercial Cu_2O and c-Cu_2O-pyS. (d) Partial current density of acetate over commercial Cu_2O and c-Cu_2O-pyS. (e) FE of acetate over commercial CuO and c-CuO-pyS. (F) Partial current density of acetate over commercial CuO and c-CuO-pyS. Error bars represent the standard deviation of 3 replicate measurements.

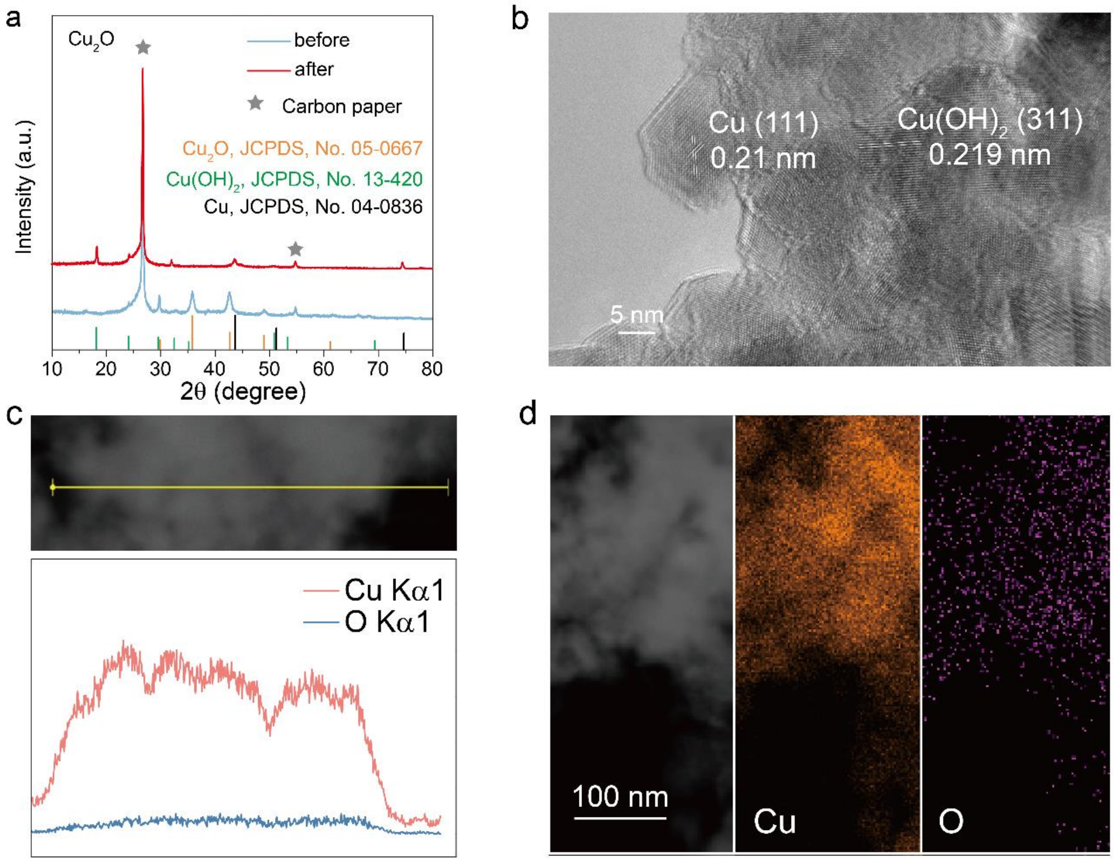

Supplementary Fig. 10 (a) XRD patterns of Cu_2O nanocubes before and after CORR. (b) TEM image of Cu_2O nanocubes after CORR. (c) EDS line scan of Cu_2O nanocubes after CORR. The top image showing the HAADF-STEM image of Cu_2O nanocubes after CORR. The bottom image showing the corresponding intensity profile of Cu and O along the line as shown in the top. (d) EDS elemental mapping images of Cu_2O nanocubes after CORR.

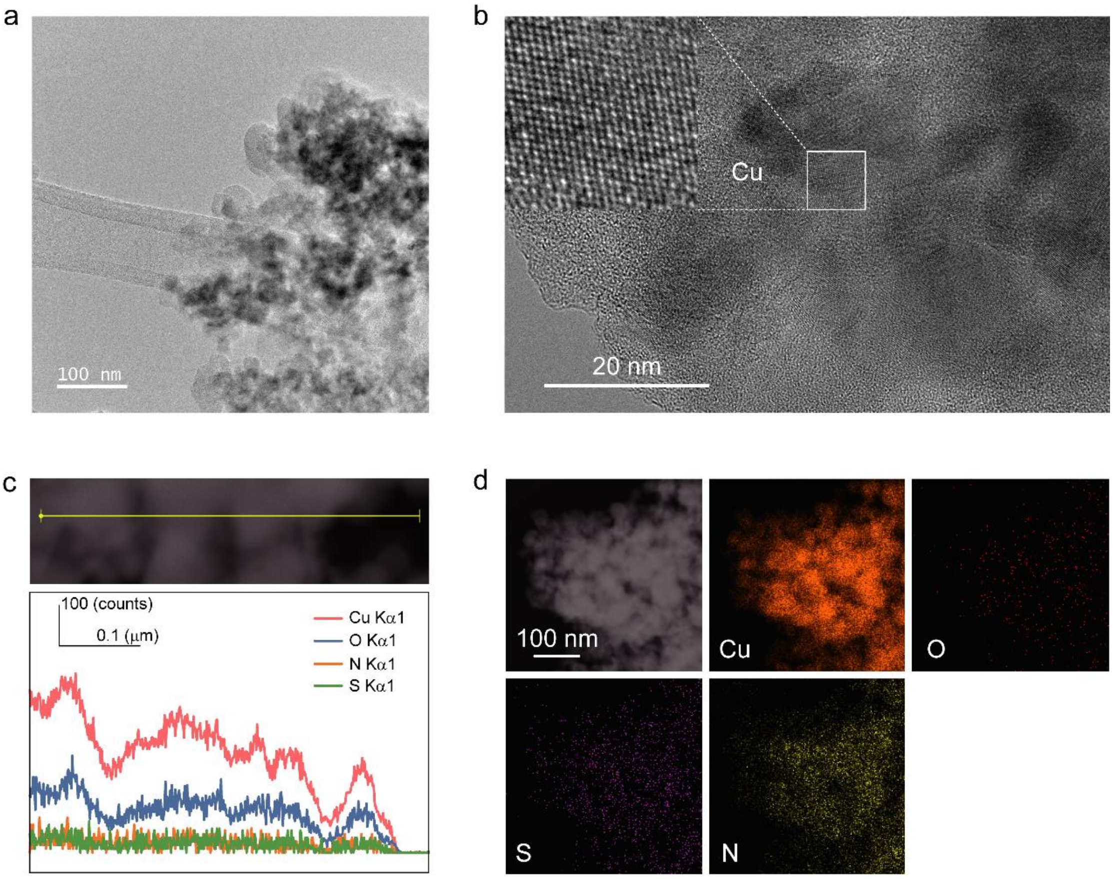

Supplementary Fig. 11 (a) XRD patterns of Cu_2O-pyS before and after CORR. (b) TEM image of Cu_2O-pyS after CORR. (c) EDS line scan of Cu_2O-pyS after CORR. The top image (scale bar: 100 nm) showing the HAADF-STEM image of Cu_2O-pyS after CORR. The bottom image showing the corresponding intensity profile of Cu and O along the line as shown in the top. (d) EDS elemental mapping images of Cu_2O-pyS after CORR.

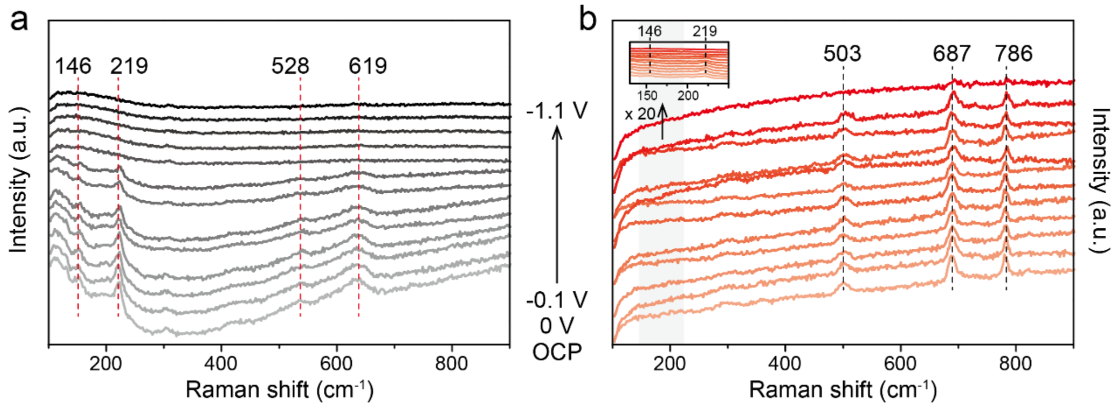

Supplementary Fig. 12 In-situ Raman spectroscopy. Raman spectra of (a) Cu_2O nanocubes and (b) Cu_2O-pyS recorded in Ar-saturated 1 M KOH solution at different applied cathodic potentials. The peaks at around 689 and 783 cm^{-1} can be assigned to Cu-S vibration.

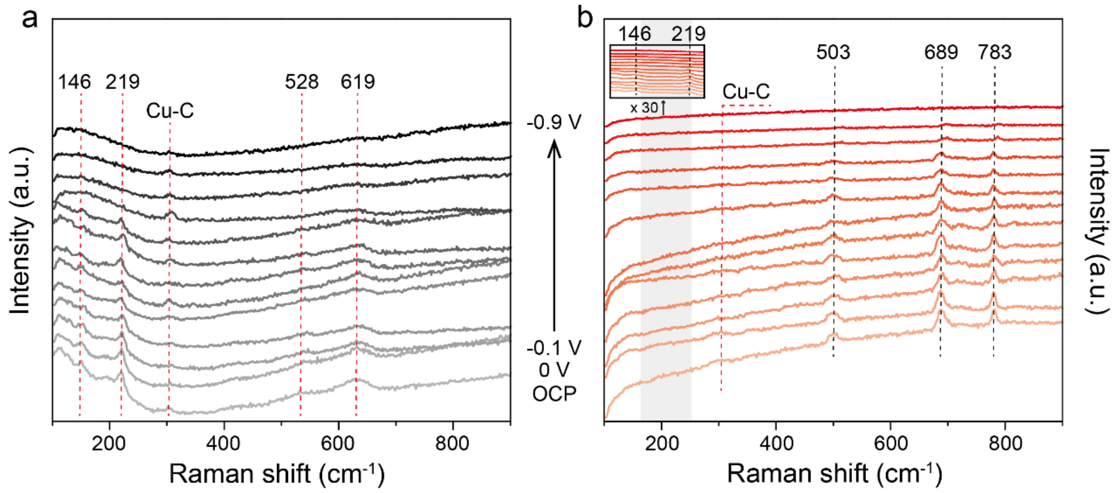

Supplementary Fig. 13 In-situ Raman spectroscopy. Raman spectra of (a) Cu_2O nanocubes and (b) Cu_2O-pyS recorded in CO-saturated 1 M KOH electrolyte at different applied cathodic potentials. The peaks at around 689 and 783 cm^{-1} can be assigned to Cu-S vibration.

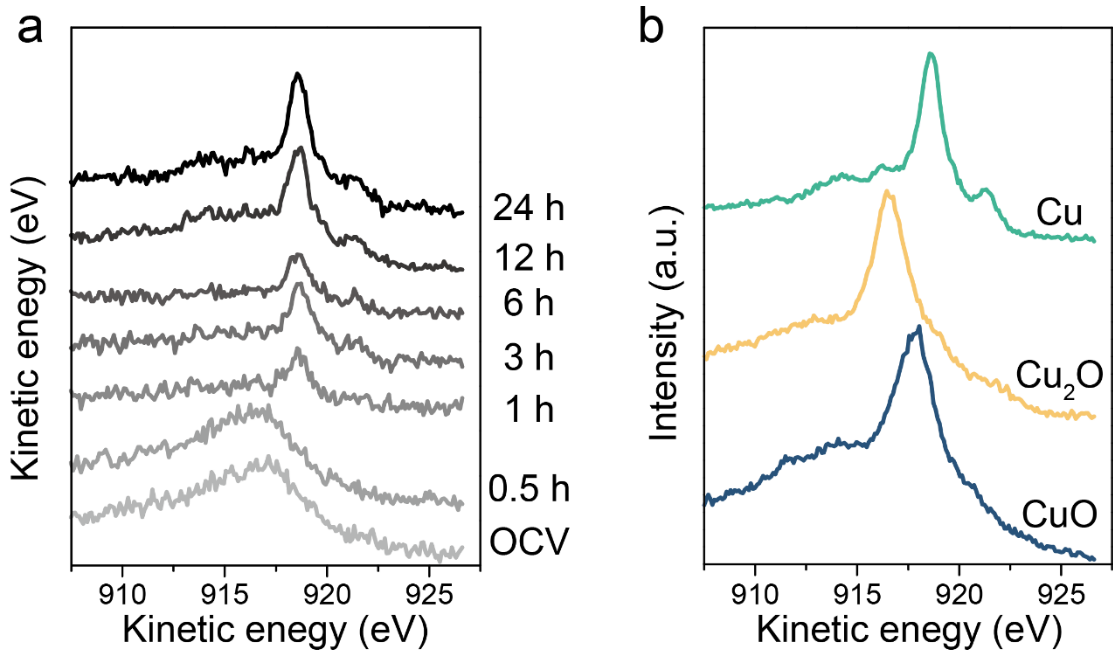

Supplementary Fig. 14 (a) Cu LMM X-ray-excited Auger electron spectra of Cu_2O nanocubes at -0.65 V vs. RHE. (b) Cu LMM X-ray-excited Auger electron spectra of various standard samples at -0.65 V vs. RHE.

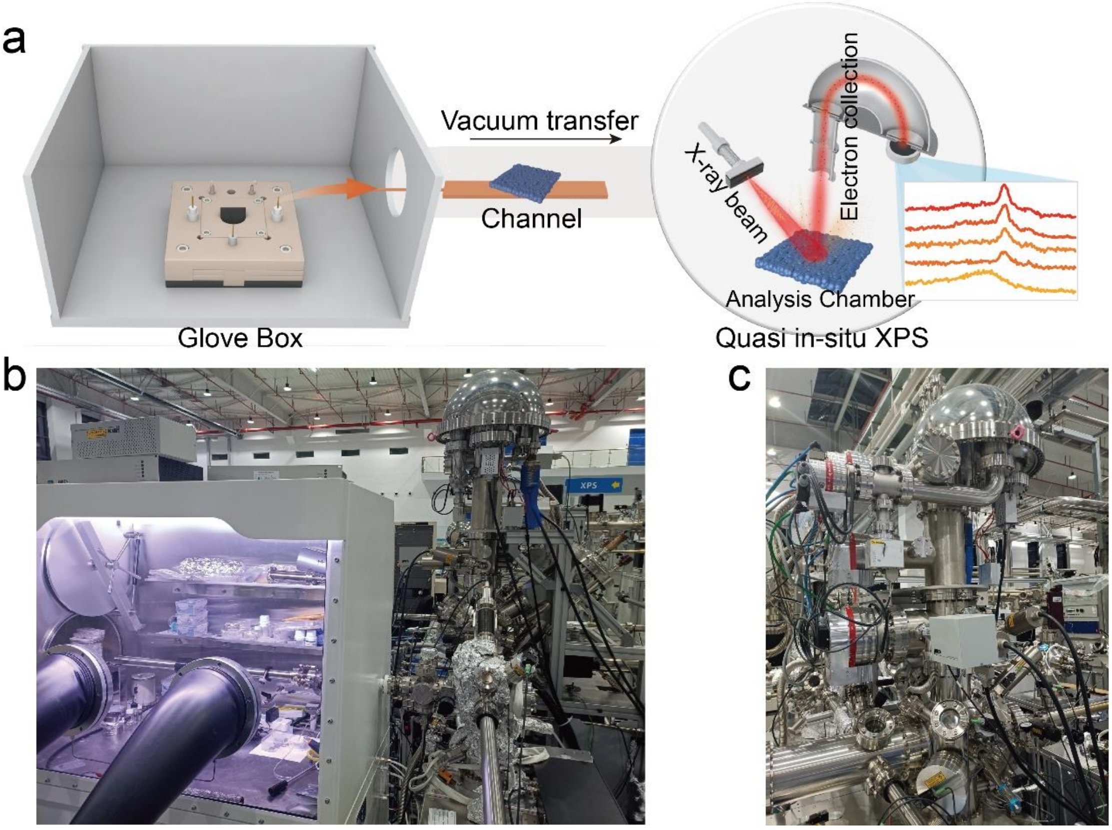

Supplementary Fig. 15 (a) Schematic illustration showing the *in-situ* XPS measurement system. (b) A digital image of the quasi *in-situ* XPS measurement system. (c) A digital image of the XPS instrument.

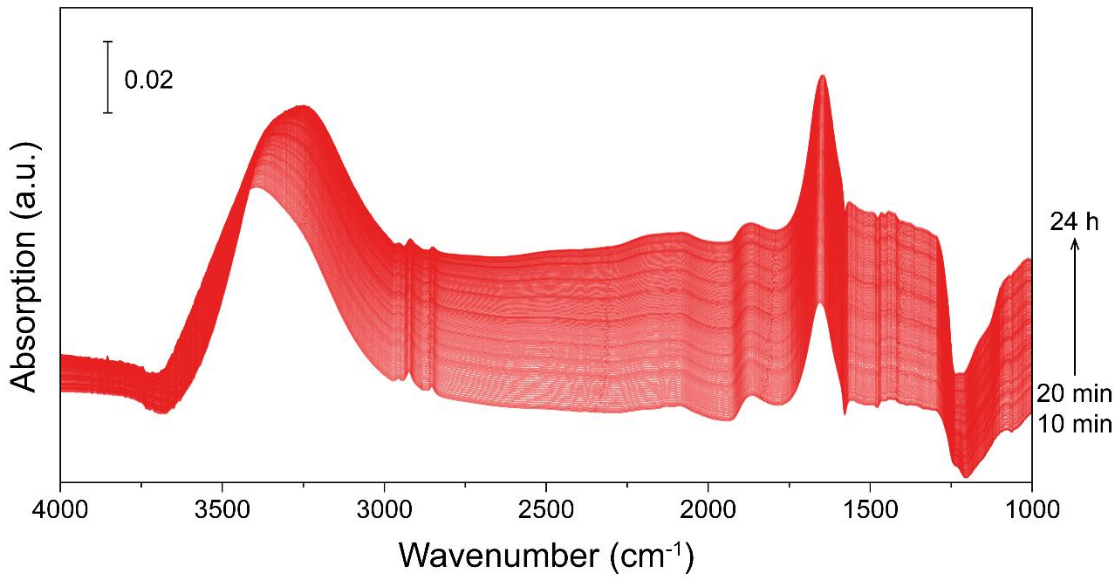

Supplementary Fig. 16 Time-resolved *in-situ* ATR-SEIRAS spectra from 1000 cm^{-1} to 4000 cm^{-1} recorded over Cu_2O-pyS in CO-saturated 0.1 M KOH solution at -0.65 V vs. RHE.

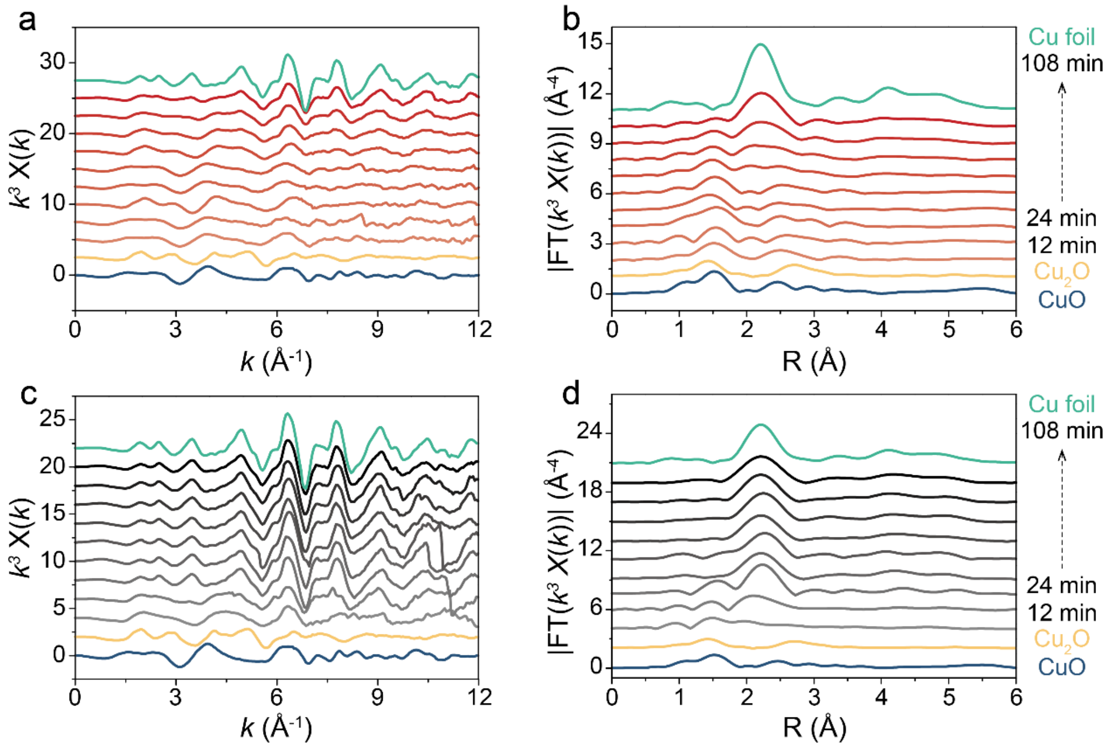

Supplementary Fig.17 The k^3-weighted EXAFS in *K*-space for Cu_2O nanocubes (a) and Cu_2O-pyS (c) measured in CO-saturated KOH solution at -0.65 V vs. RHE. The corresponding Fourier transform of EXAFS curves for Cu_2O nanocubes (b) and Cu_2O-pyS (d) measured in CO-saturated KOH solution at -0.65 V vs. RHE.

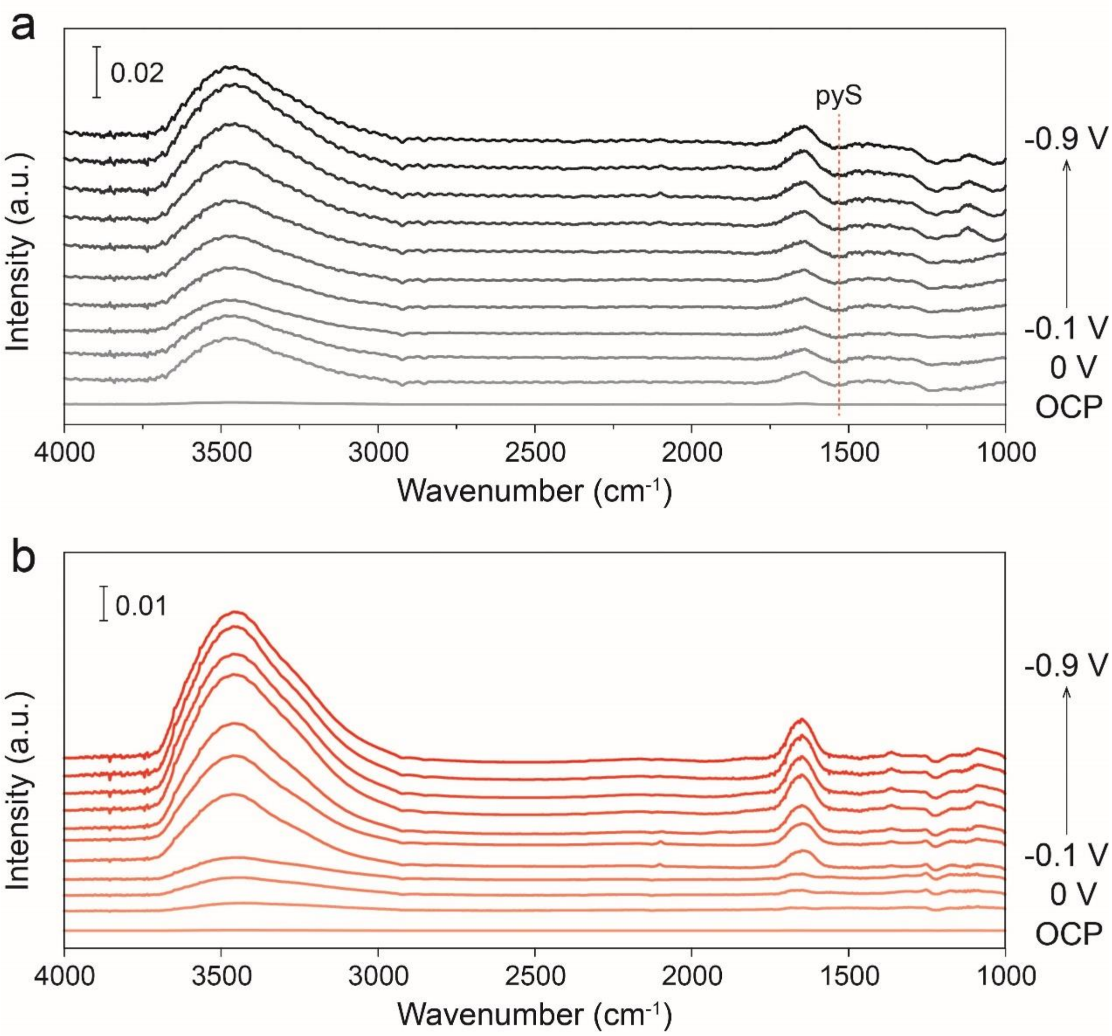

Supplementary Fig. 18 In-situ ATR-SEIRAS spectra recorded over Cu_2O (a) and Cu_2O (b) nanocubes in Ar-saturated KOH solution at different applied cathodic potentials.

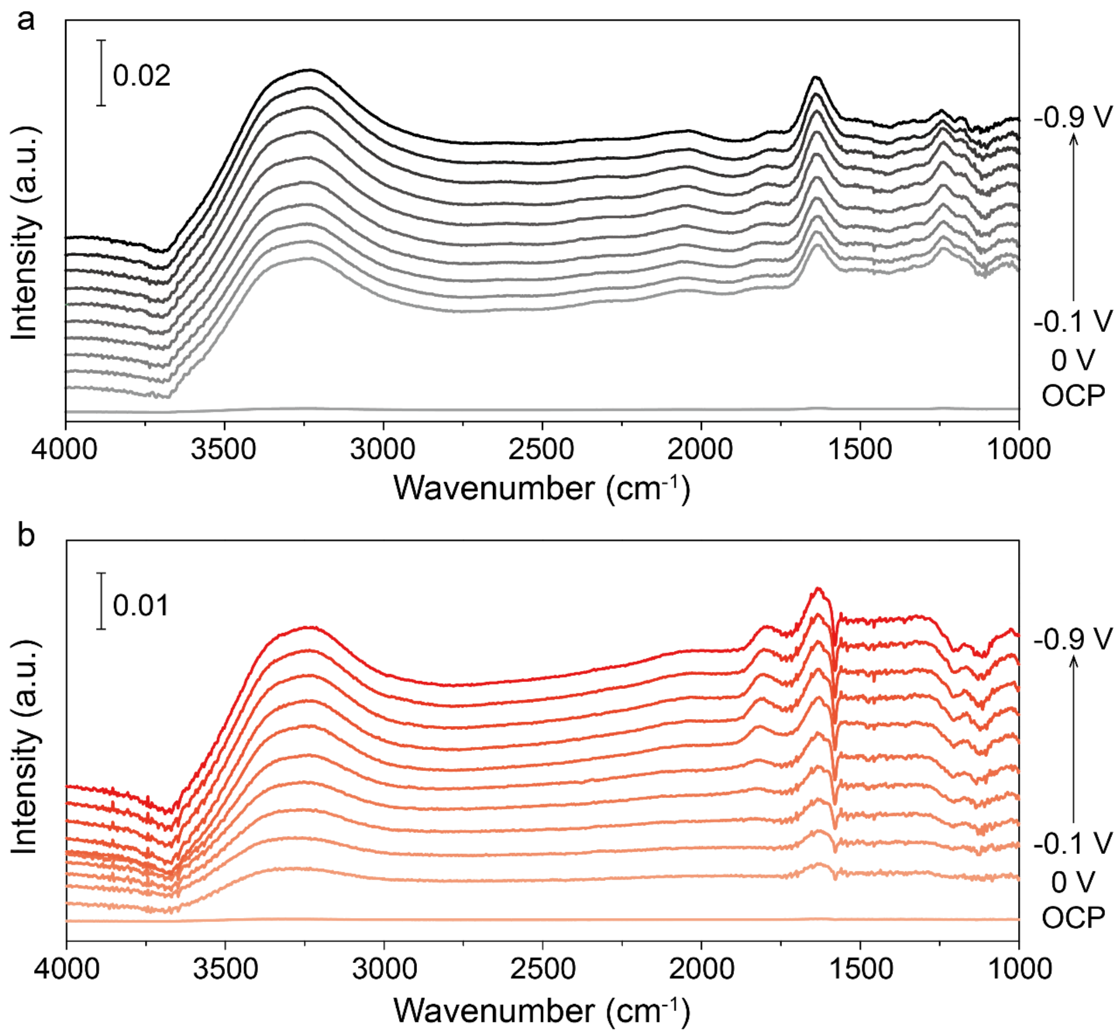

Supplementary Fig. 19 (a) In-situ ATR-SEIRAS spectra recorded over Cu_2O in CO-saturated 0.1 M KOH solution at different applied cathodic potentials. (b) *In-situ* ATR-SEIRAS spectra recorded over Cu_2O-pyS in CO-saturated 0.1 M KOH solution at different applied cathodic potentials.

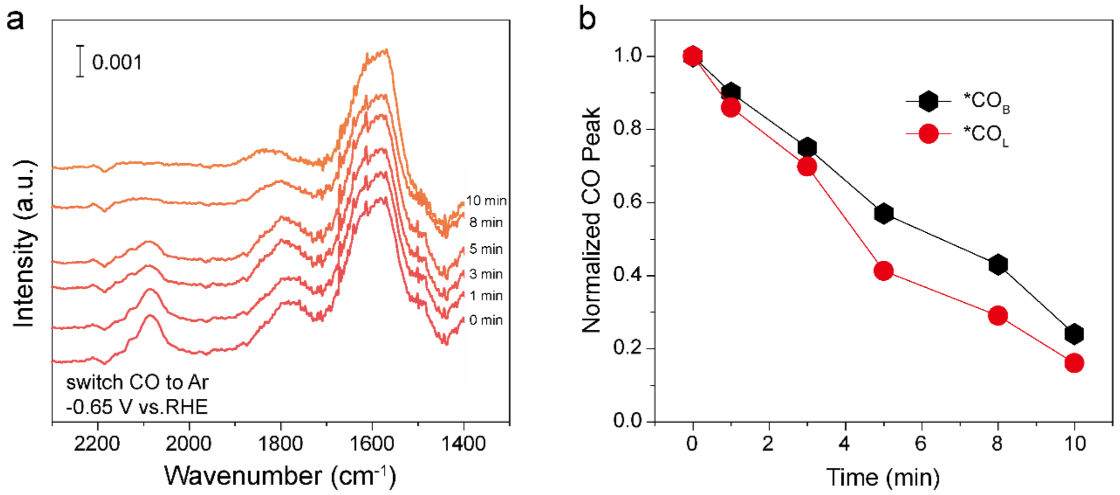

Supplementary Fig. 20 (a) In-situ ATR-SEIRAS spectra on Cu_2O-pyS as a function of time. (b) Temporal evolution of normalized peak area of $*CO_L$ and $*CO_B$. Note: To confirm whether CO was involved in C-C coupling to acetate, the intensities of CO_B and CO_L were monitored using *in-situ* ATR-SEIRAS. The *in-situ* ATR-SEIRAS spectra were recorded during continuous CO purging until the CO peaks reached a virtually constant state. Upon reaching the maximum CO peak, the purge gas was switched from CO to Ar and maintained at -0.65 V_{RHE}. At the moment of switching gas, the ATR-SEIRAS spectra were recorded. The results show that both the $*CO_L$ and $*CO_B$ species are consumed under Ar atmosphere at -0.65 V vs. RHE, suggesting that both $*CO_L$ and $*CO_B$ are reactive species in CORR towards acetate formation.

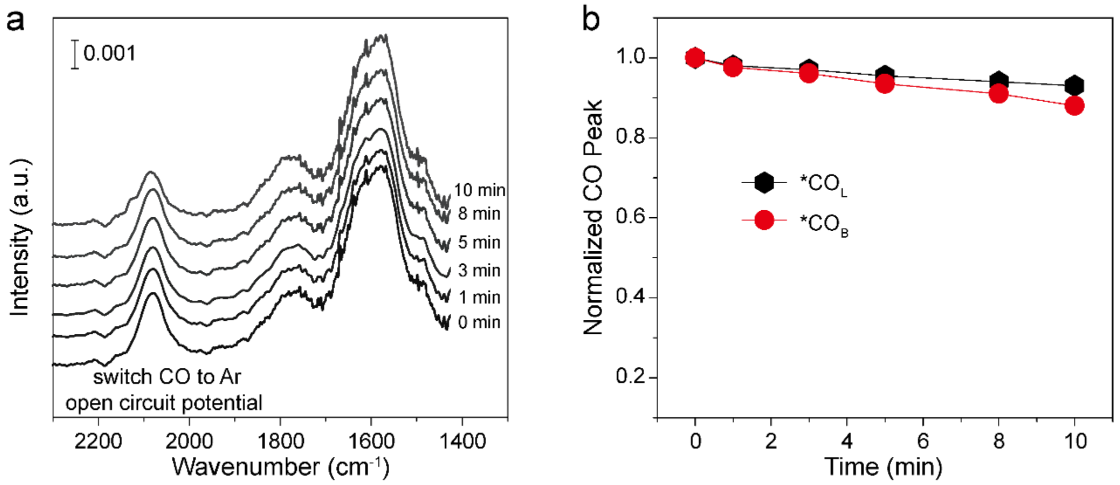

Supplementary Fig. 21 (a) In-situ ATR-SEIRAS spectra on Cu_2O-pyS as a function of time. (b) Temporal evolution of normalized peak area of $*CO_L$ and $*CO_B$. Note: To rule out the possibility of *CO desorption caused by Ar purge (rather than its consumption) that led to decrease in *CO peak in Supplementary Figure 19, *in-situ* ATR-SEIRAS spectra were recorded during continuous CO purging until the CO peaks reached a virtually constant state. Upon reaching the maximum CO peak, the purge gas was switched from CO to Ar, and the applied cathodic potential was stopped. The results showed that Ar purge played a negligible influence on the decrease of CO peaks. Besides, at a low coverage of CO on catalyst's surface, CO tends to form bridge structure with neighboring atoms or molecules owing to the fact that bridge adsorption of CO generally exhibits greater strength compared to linear adsorption. However, as the coverage of CO increases, it transforms to linear adsorption where individual CO molecules bind directly to metal sites. Interestingly, 4-mercatopyridine modification on the surface of Cu can significantly impact the adsorption properties of CO. The inclusion of 4-mercatopyridine leads to an accelerated consumption of CO, which reduces its overall coverage on the catalyst's surface. Consequently, the remaining adsorbed CO species exhibit a higher inclination towards bridge adsorption.

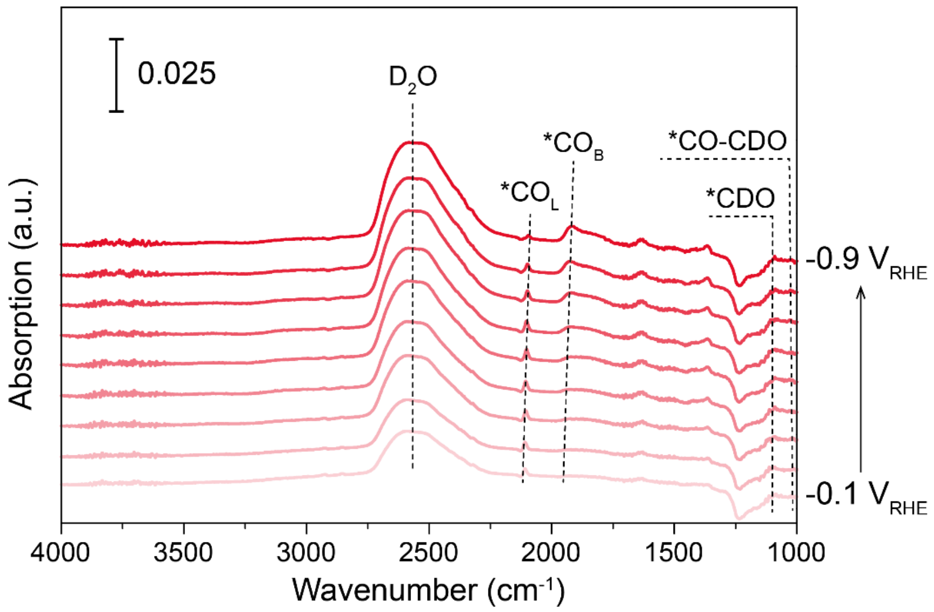

Supplementary Fig. 22 In-situ ATR-SEIRAS spectra recorded in a potential window from -0.1 to -0.9 V vs. RHE over Cu_2O-pyS in 0.1 M KOD.

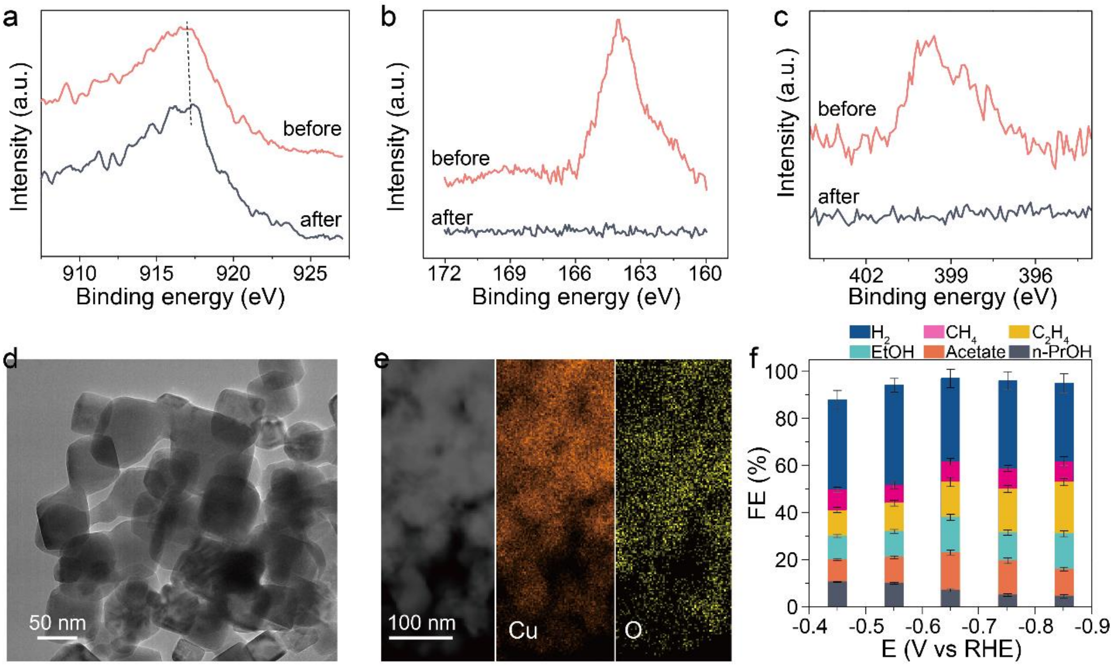

Supplementary Fig. 23 High-resolution (a) Cu 2p (b) S 2p XPS and (c) N 1s XPS spectra of Cu_2O nanocubes before and after the Ar plasma treatment. (d) TEM image of Cu_2O nanocubes after the Ar plasma treatment. (e) EDS elemental mapping images of Cu_2O nanocubes after the Ar plasma treatment. (f) FE of products over Cu_2O nanocubes after the Ar plasma treatment. Error bars represent the standard deviation of 3 replicate measurements.

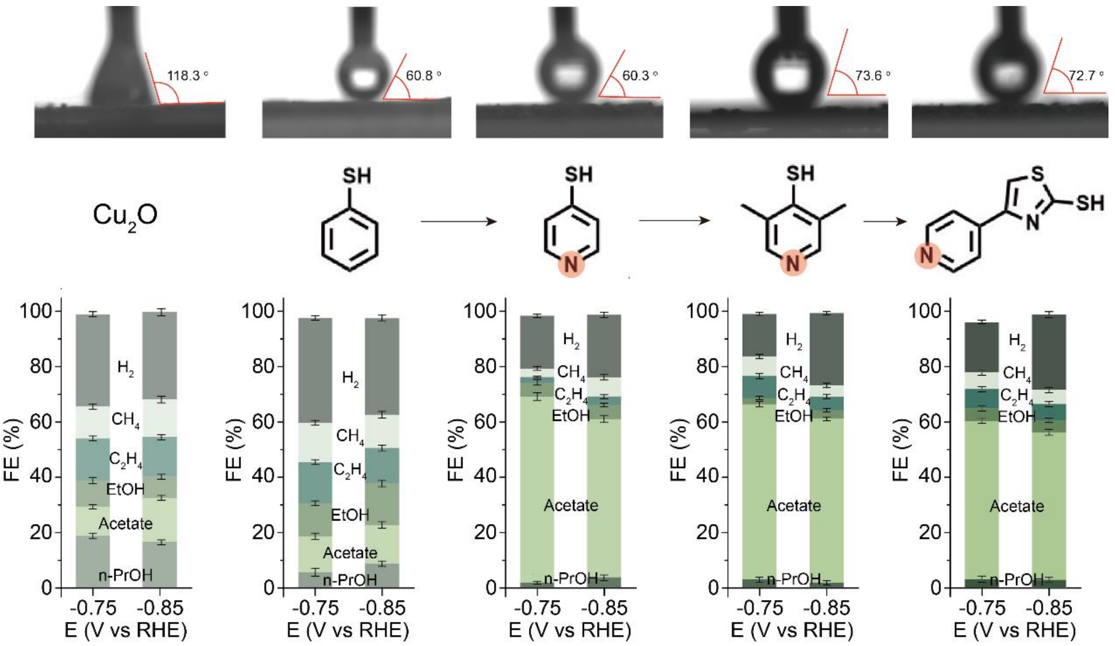

Supplementary Fig. 24 Different molecules used to modify the surface of Cu_2O. The corresponding contact angle (top) and CORR performance (down). Error bars represent the standard deviation of 3 replicate measurements.

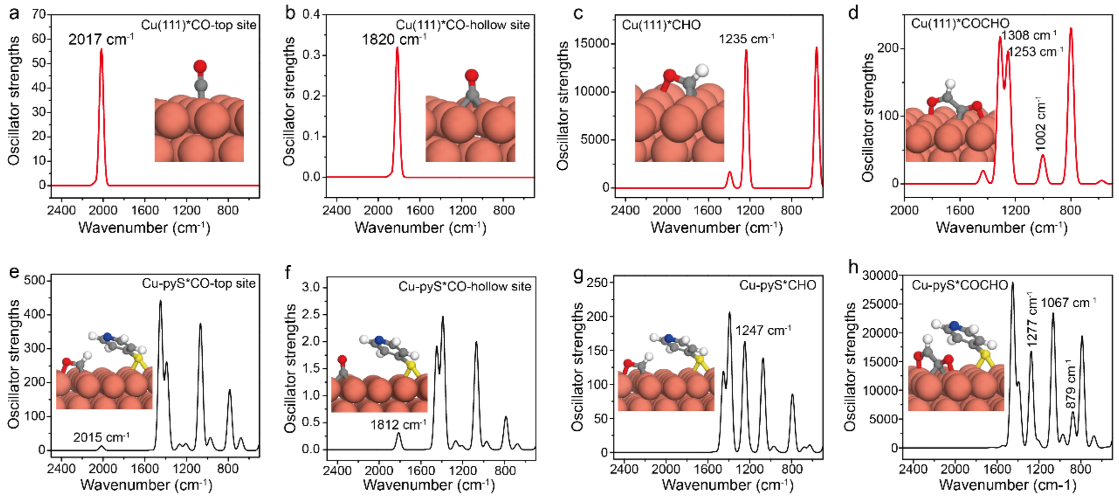

Supplementary Fig. 25 Simulated IR peaks of important intermediates. (a) *CO simulated peak over top site of Cu (111). (b) *CO simulated peak over hollow site of Cu (111). (c) *CHO simulated peak over Cu (111). (d) *COCHO simulated peak over Cu (111). (e) *CO simulated peak over top site of Cu-pyS. (f) *CO simulated peak over hollow site of Cu-pyS. (g) *CHO simulated peak over Cu-pyS. (h) *COCHO simulated peak over Cu-pyS.

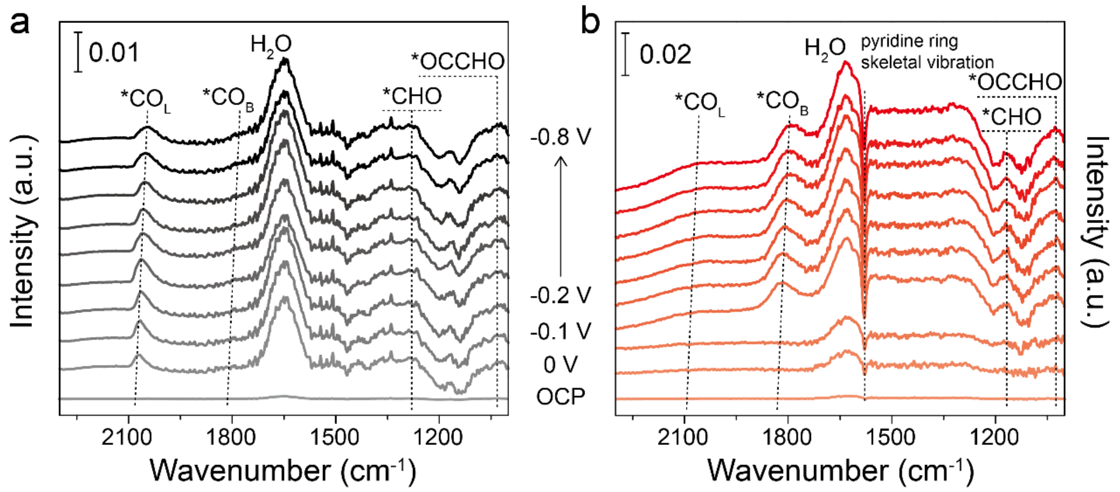

Supplementary Fig. 26 (a) *In-situ* ATR-SEIRAS spectra recorded over commercial Cu_2O in CO-saturated 0.1 M KOH solution at different applied cathodic potentials. (b) *In-situ* ATR-SEIRAS spectra recorded over c-Cu_2O-pyS in CO-saturated 0.1 M KOH solution at different applied cathodic potentials.

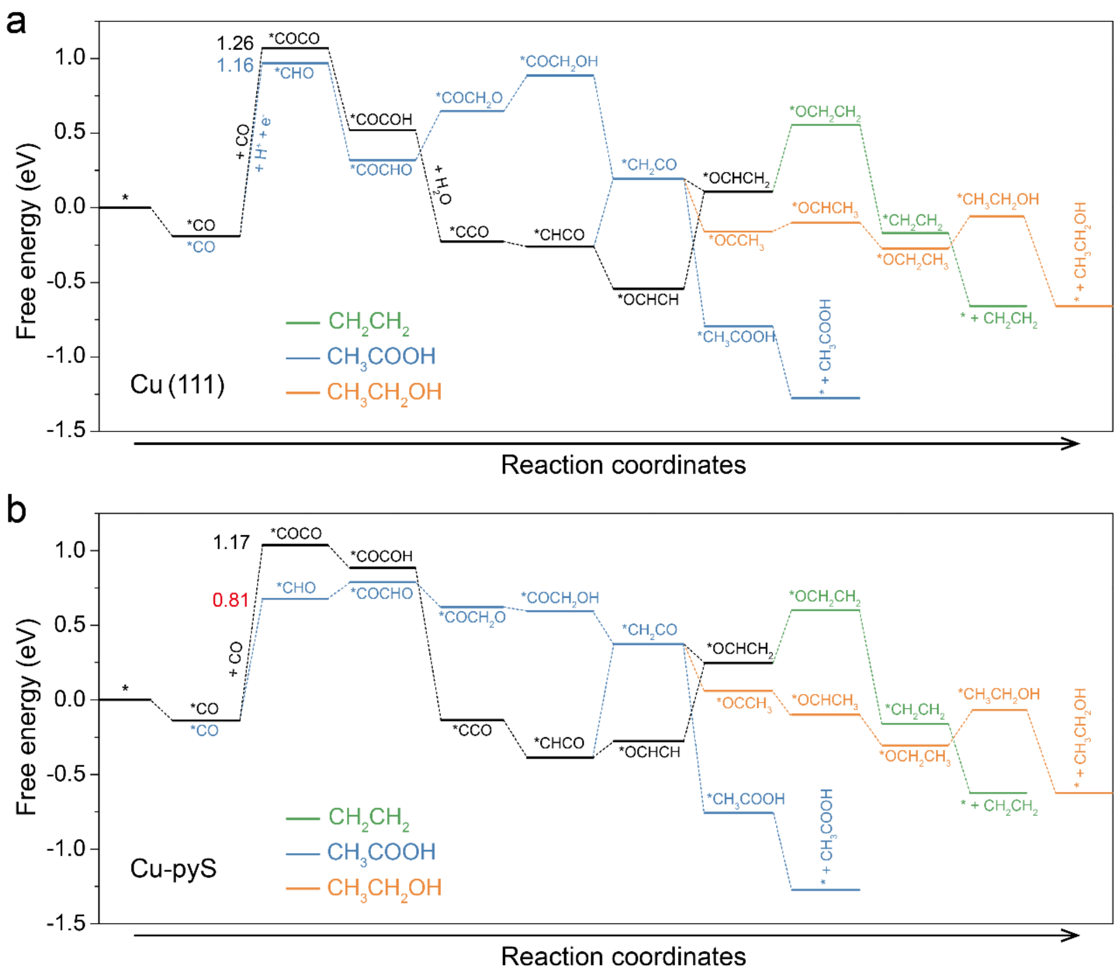

Supplementary Fig. 27 Free energy diagram of CORR over Cu_2O (a) and Cu_2O-pyS (b).

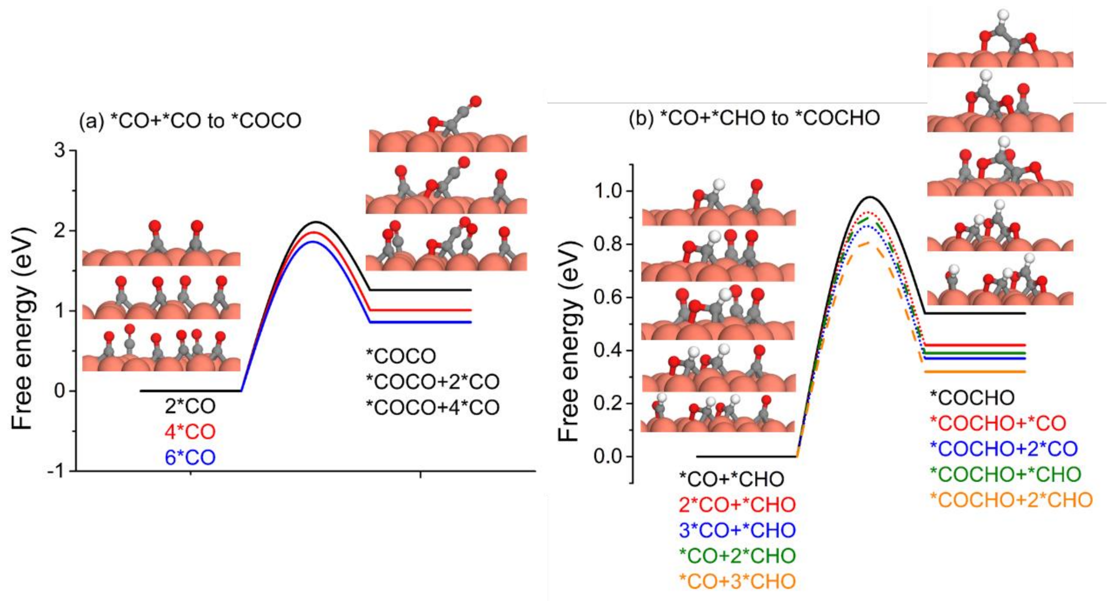

Supplementary Fig. 28 (a) Free energy diagram showing the formation of *CO + *CO to *COCO (b) *CO + *CHO to *COCHO for different coverages of *CO and *CHO.

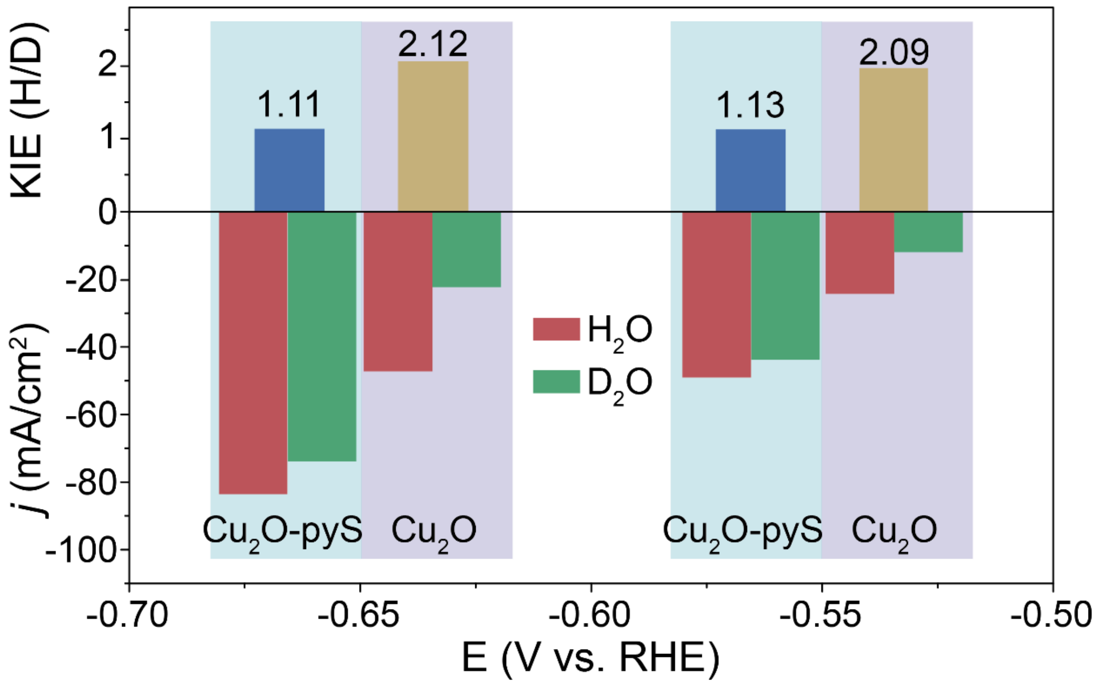

Supplementary Fig. 29 KIE of H/D in CORR to acetate at -0.55 V and -0.65 V vs. RHE over Cu_2O nanocubes and Cu_2O-pyS.

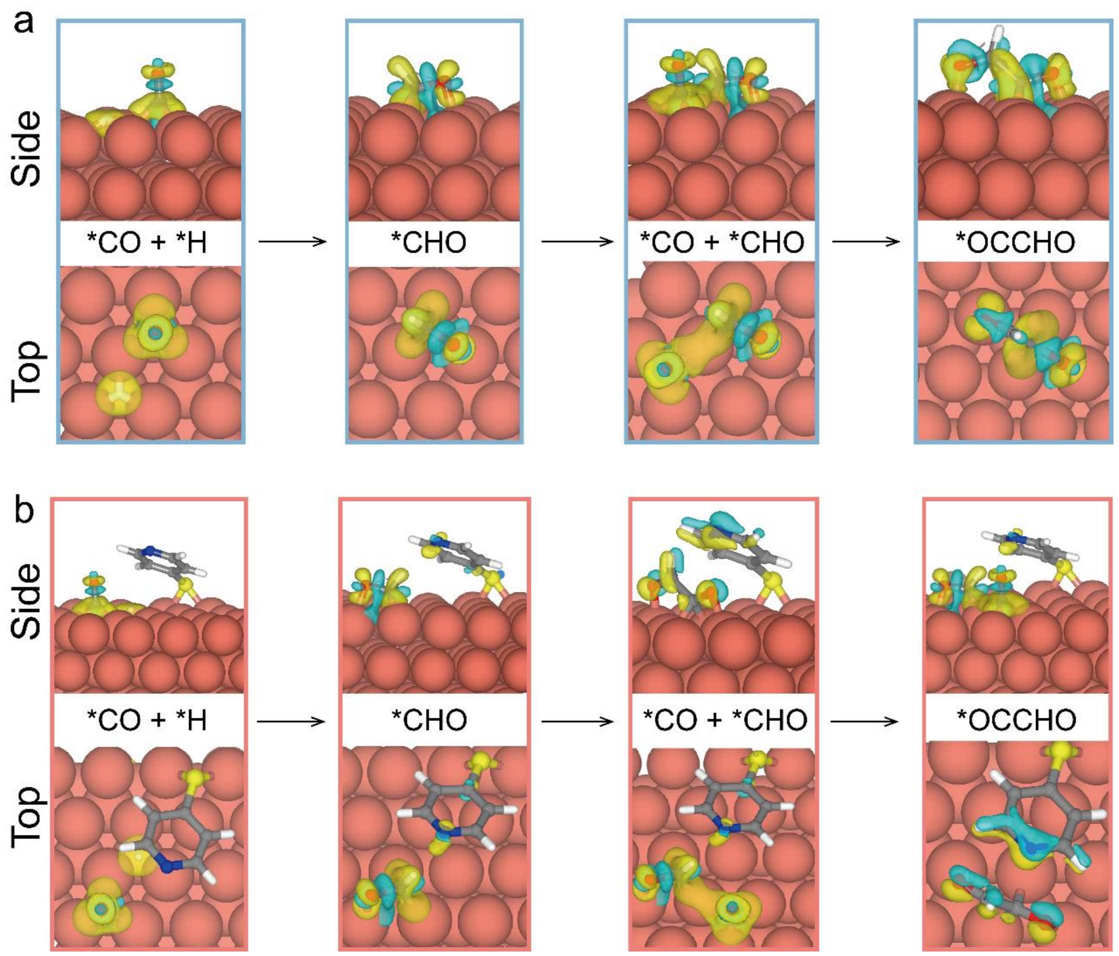

Supplementary Fig. 30 Differential charge density for various important intermediates over (a) Cu_2O and (b) Cu_2O-pyS. Yellow and blue regions represent electron accumulation and depletion, respectively.

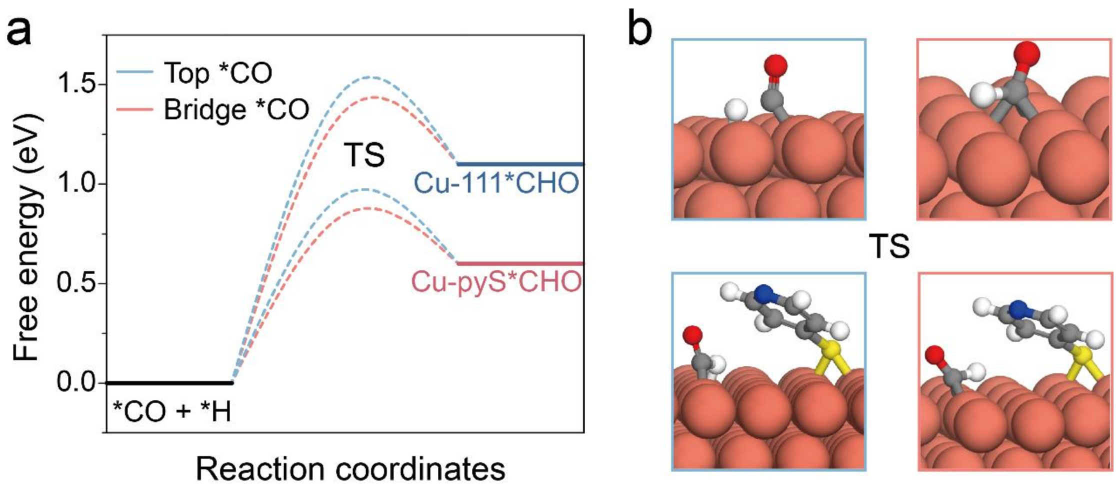

Supplementary Fig. 31 (a) Free energy diagram of top *CO and bridge *CO hydrogenation over Cu and Cu-pyS. (b) The transition states of the hydrogenation step.

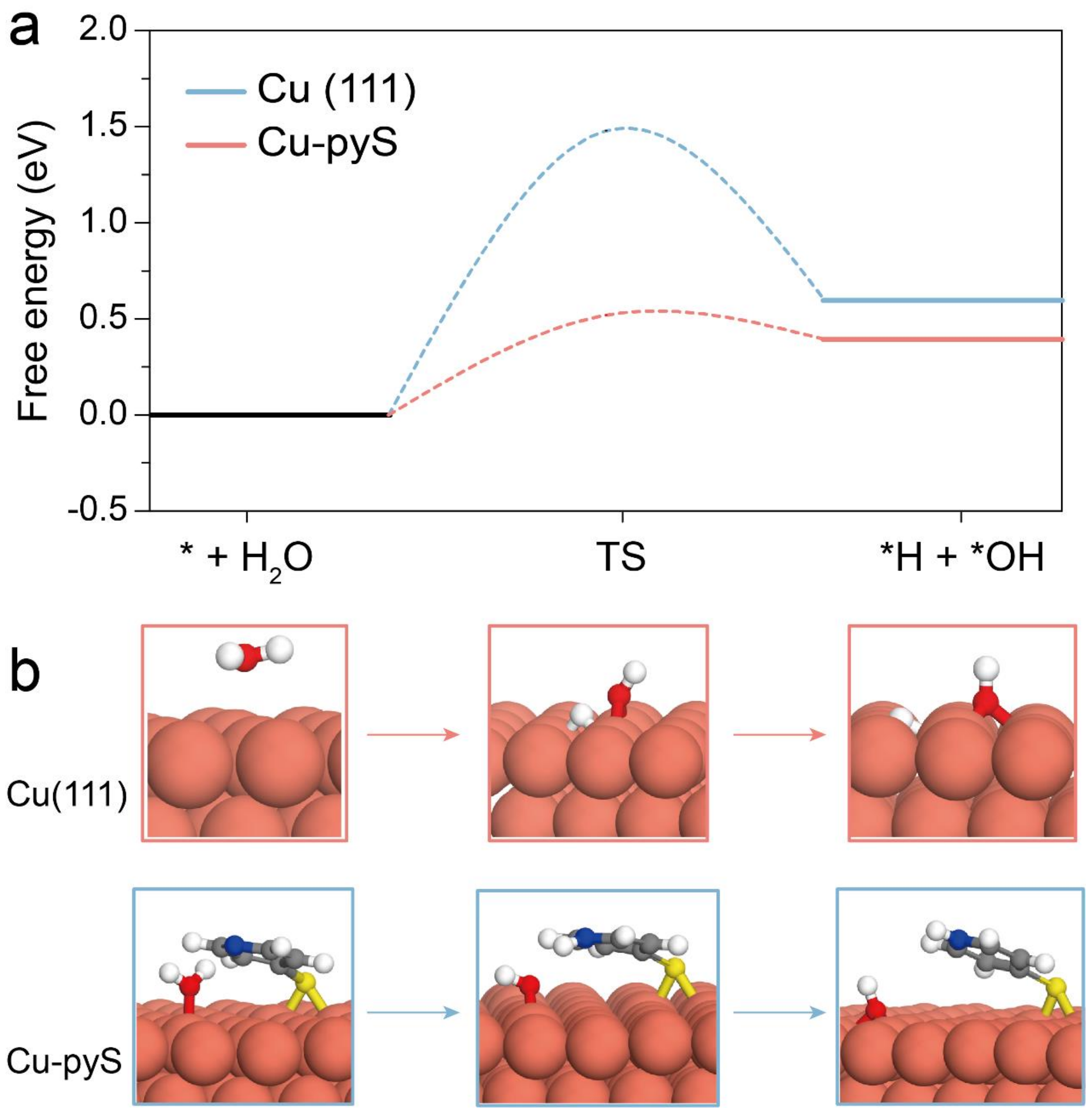

Supplementary Fig. 32 (a) Free energy diagram of water dissociation over Cu (111) and Cu-pyS. (b) The side view images of water dissociation process over Cu (111) and Cu-pyS.

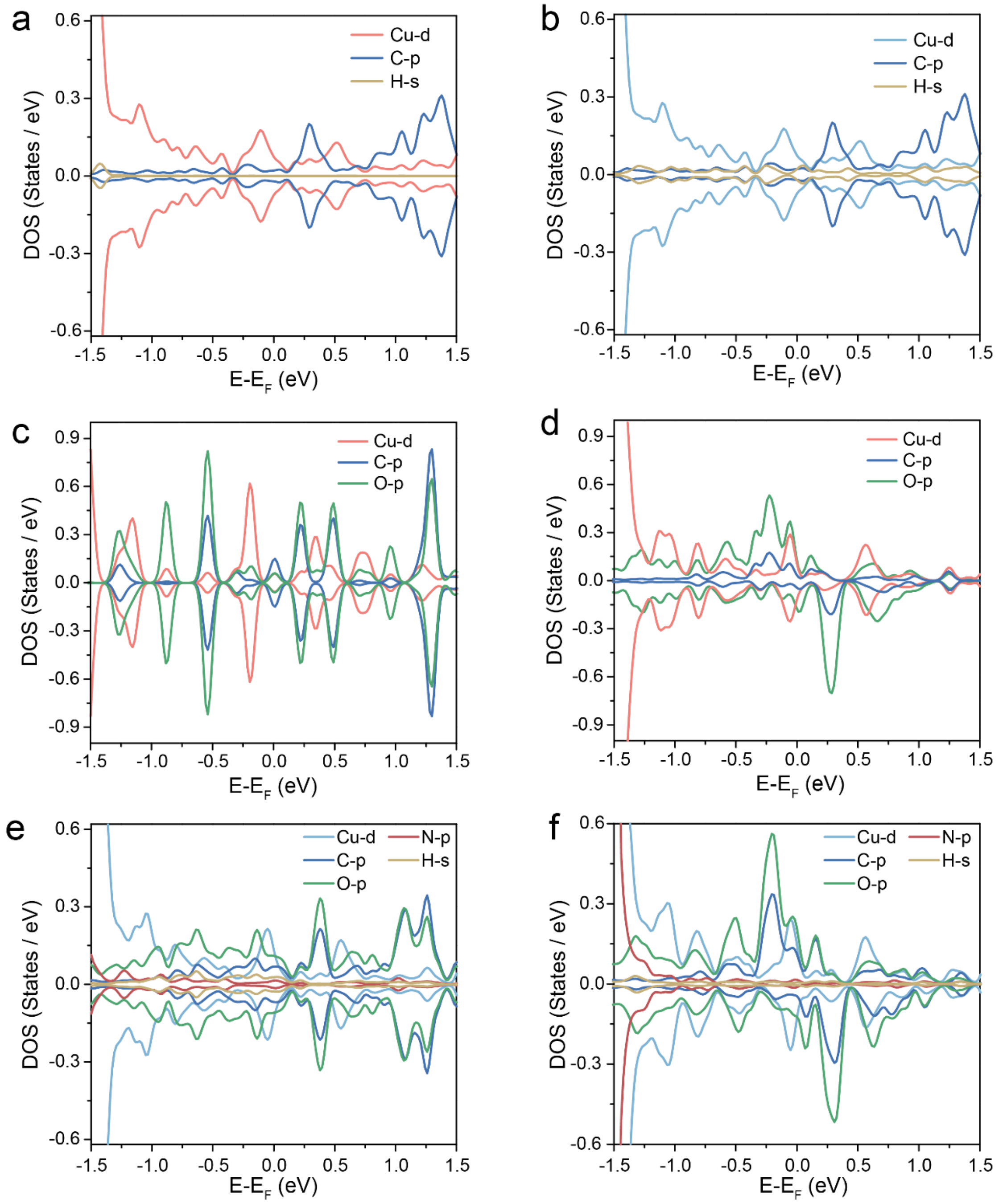

Supplementary Fig. 33 *CO + *H over Cu_2O (a) and Cu_2O-pyS (b). *CHO over Cu_2O (c) and Cu_2O-pyS (d). *OCCHO over Cu_2O (e) and Cu_2O-pyS (f).

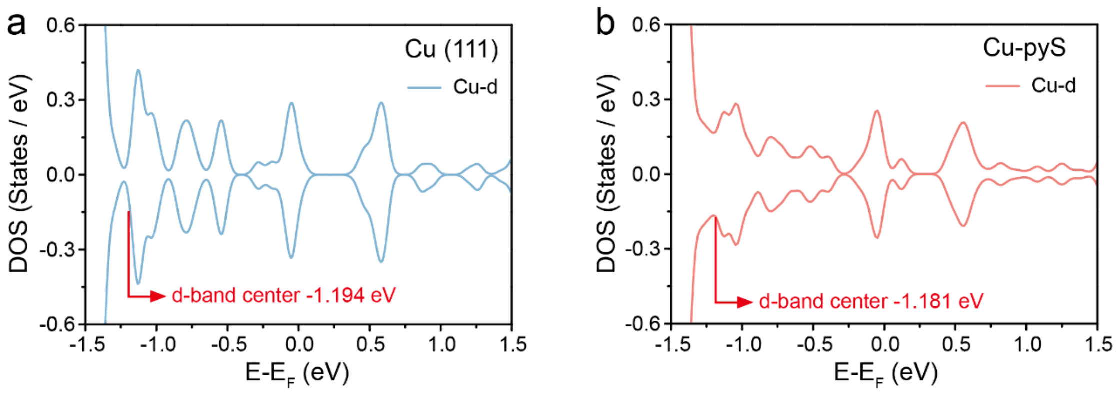

Supplementary Fig. 34 D-band center of Cu_2O (a) and Cu_2O-pyS (b).

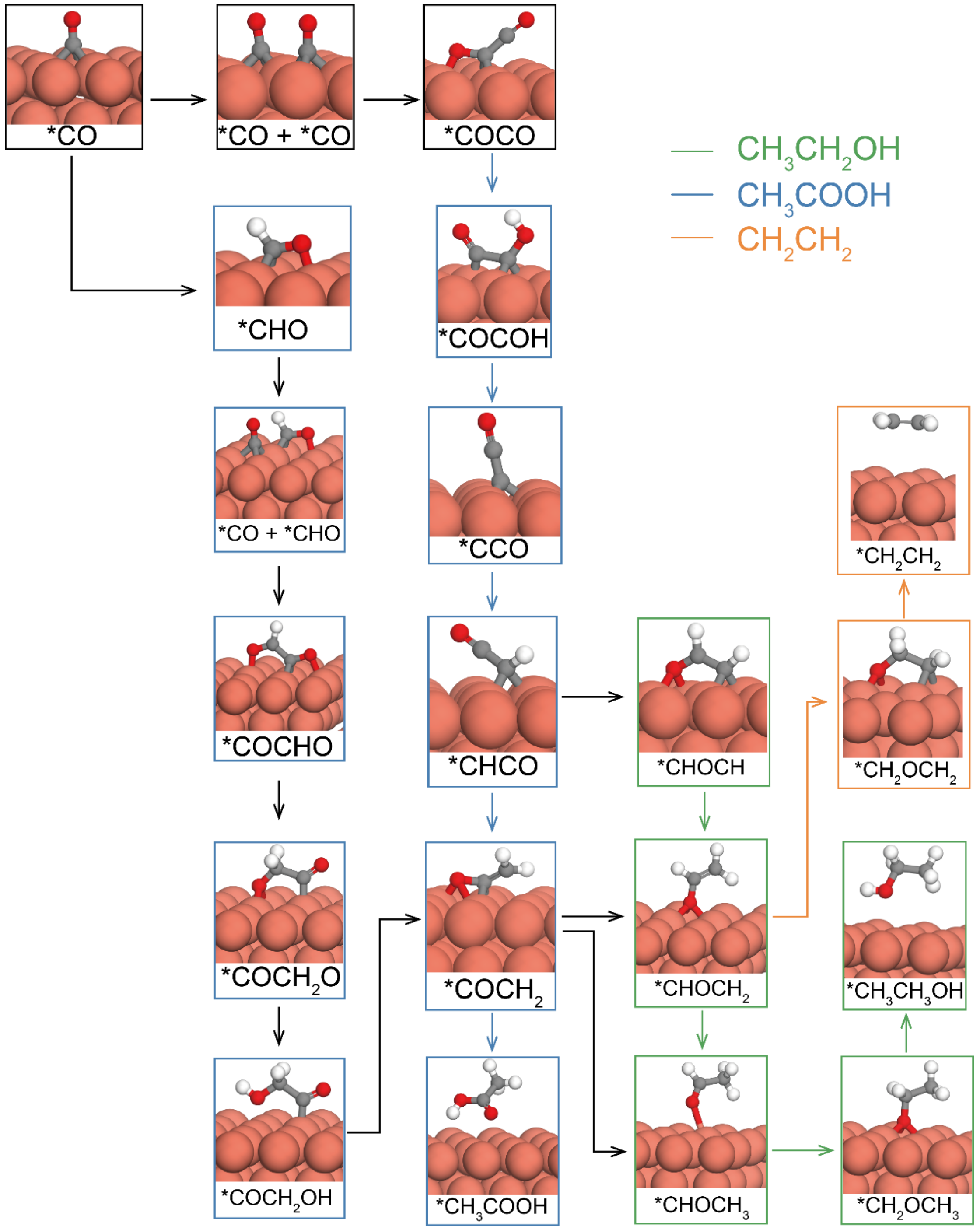

Supplementary Fig. 35 The reaction pathway to produce CH_3CH_2OH, CH_3COOH and CH_2CH_2 over Cu_2O.

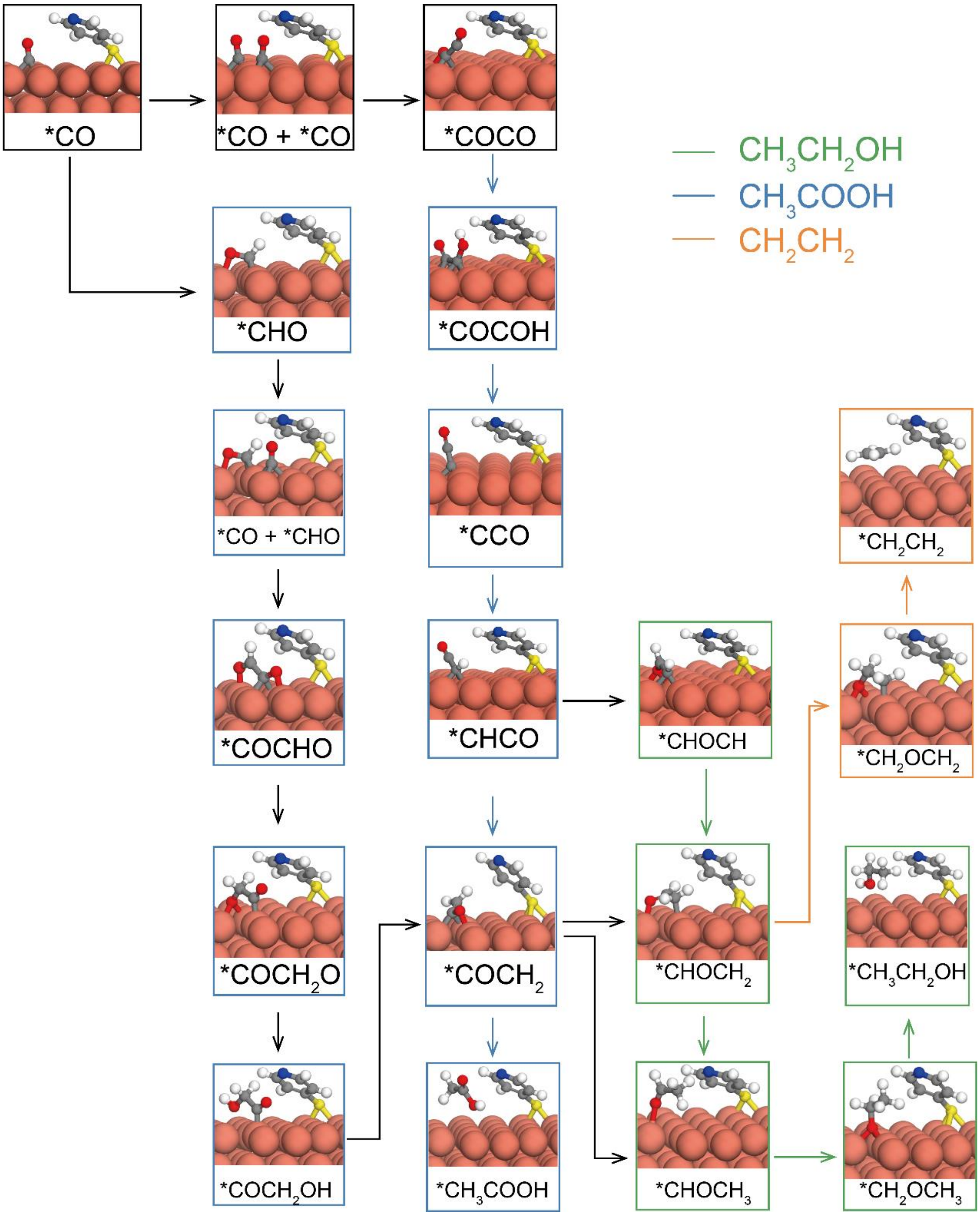

Supplementary Fig. 36 The reaction pathway to produce CH_3CH_2OH, CH_3COOH and CH_2CH_2 over Cu_2O-pyS.
